# Predictive validity of the Stopping Elderly Accidents, Deaths & Injuries (STEADI) program fall risk screening algorithms among community-dwelling Thai elderly

**DOI:** 10.1186/s12916-022-02280-w

**Published:** 2022-03-14

**Authors:** Sriprapa Loonlawong, Weerawat Limroongreungrat, Thanapoom Rattananupong, Kamonrat Kittipimpanon, Wanvisa Saisanan Na Ayudhaya, Wiroj Jiamjarasrangsi

**Affiliations:** 1 Regional Health Promotion Center 9 Nakhon Ratchasima, Department of Health, 177 Moo.6 Khok Kruat Sub-district, Muang District, Nakhon Ratchasima, 30280 Thailand; 2 College of Sports Science and Technology, Mahidol University, 999 Phuttamonthon 4 Road, Salaya, Phuttamonthon, Nakhonpathom 73170 Thailand; 3 Department of Preventive and Social Medicine, Faculty of Medicine, Chulalongkorn University, Rama IV Road, Pathumwan, Bangkok, 10330 Thailand; 4 Faculty of Medicine Ramathibodi Hospital, Mahidol University, 270 Rama VI Road, Ratchathewi, Bangkok, 10400 Thailand; 5 Department of Community Public Health, School of Public Health, Walailak University, 222 Thaiburi Sub-district, Thasala District, 80160 Nakhon Si Thammarat, Thailand

**Keywords:** Predictive validity, STEADI, Fall risk screening algorithm, Elderly, Community

## Abstract

**Background:**

Fall risk screening using multiple methods was strongly advised as the initial step for preventing fall. Currently, there is only one such tool which was proposed by the U.S. Centers for Disease Control and Prevention (CDC) for use in its Stopping Elderly Accidents, Death & Injuries (STEADI) program. Its predictive validity outside the US context, however, has never been investigated. The purpose of this study was to determine the predictive validity (area under the receiver operating characteristic curve: AUC), sensitivity, and specificity of the two-step sequential fall-risk screening algorithm of the STEADI program for Thai elderly in the community.

**Methods:**

A 1-year prospective cohort study was conducted during October 2018–December 2019. Study population consisted of 480 individuals aged 65 years or older living in Nakhon Ratchasima Province, Thailand. The fall risk screening algorithm composed of two serial steps. **Step 1** is a screening by the clinician’s 3 key questions or the Thai Stay Independent brochure (Thai-SIB) 12 questions. **Step 2** is a screening by 3 physical fitness testing tools including Time Up and Go test (TUG), 30-s Chair Stand, and 4-stage balance test. Participants were then followed for their fall incidents. Statistical analyses were conducted by using Cox proportional hazard model. The AUC, sensitivity, specificity, and other relevant predictive validity indices were then estimated.

**Results:**

The average age of the participants was 73.3 ± 6.51 years (range 65–95 years), and 52.5% of them were female. The screening based on the clinician’s 3 key questions in Step 1 had a high AUC (0.845), with the sensitivity and specificity of 93.9% (95% CI 88.8, 92.7) and 75.0% (95% CI 70.0, 79.6), respectively. Appropriate risk categorization however differed slightly from the original STEADI program.

**Conclusions:**

With some modification, the fall risk screening algorithm based on the STEADI program was applicable in Thai context.

**Supplementary Information:**

The online version contains supplementary material available at 10.1186/s12916-022-02280-w.

## Background

Falling is a major threat to the elderly’s quality of life, often causing a decline in self-care ability and social activities. An estimated 646,000 elderly people around the world die from falls each year [1]. Falls account for 40% of all injurious deaths [2]. In Thailand and worldwide, falls are the second leading cause of injury death after road traffic accidents. Non-fatal falls resulted in minor to very severe injuries, with some of the fallers having disability and premature death [2, 3]. The direct medical costs for falls total nearly $30 billion annually [4].

A fall prevention program comprising screening for individual’s risk factors together with risk factor management is the most effective way to prevent accidental falls [5–8]. If the program is managed properly, it can reduce the rate of falls by 24% [8]. Therefore, a screening tool for fall risk is the first key and should be sensitive and specific in predicting fall risk as well as having the ability to identify the cause or risk factor(s) of fall. While a number of fall risk screening tools do exist currently, no information has clearly identified which tools are best [9]. There are only recommendations mentioning that since there is no single tool showing sufficiently high predictive validity, multiple tools should be used in combination without specific detail on the suggested combined procedure [10–12].

Currently, there is only one multi-tool fall risk screening algorithm based on sequential test, which was proposed by the U.S. Centers for Disease Control and Prevention (CDC) for use in its Stopping Elderly Accidents, Death & Injuries (STEADI) program [4, 5, 13–15]. The first step identifies high fall-risk elderly population by using both a short self-assessment questionnaire “Stay Independent” brochure (SIB) comprising 12 questions and 3 key questions asked by clinicians about past fall history. Only those with the scores ≥ 4 on the Stay Independent brochure or “Yes” answer to any key question were considered at-risk of fall and would be further screened in the second step with a more sophisticated method such as physical fitness tests including Timed Up and Go (TUG) test, 30-S Chair Stand, and the 4-Stage Balance test. From these two steps, the elderly can be classified as having low, medium and high risk of fall. Those with high risk are further assessed for multiple risk factors for risk management. STEADI is an evidence-based intervention program that offers a coordinated approach to implementing the professionals’ clinical practice guidelines for fall prevention [16]. Its screening algorithm had good psychometric properties including concurrent and predictive validity [17–19], although improvement is needed [20]. For example, the proposed screening guidelines for clinician’ 3 key questions combination with TUG or the application of a self-assessment with TUG are lacking predictive accuracy measurement [19]. In addition, the generalizability and validity of the STEADI screening algorithm have never been examined outside the USA, especially in Asian context.

Thailand’s Ministry of Public Health (MOPH) has implemented the TUG as a fall risk screening tool for the elderly in community [21]. Despite being one of the most evidence-supported and an initial screening tool for assessing fall risks, TUG is not recommended to be used as a single screening tool [10, 12]. Therefore, we have developed multiple-tool screening algorithms for elderly fall-risk in Thailand. The algorithms account for local practicality, i.e., limited resources, and a disproportion between healthcare manpower and the rapidly increasing number of elderly in Thailand’ primary care setting where the fall risk screening is performed.

To examine the applicability of the US CDC’s STEADI screening algorithm in Thailand. This study aimed to determine the predictive validity (area under the receiver operating characteristic curve or AUC, sensitivity, specificity, positive predictive value or PPV, and negative predictive value or NPV) of the two-step sequential fall-risk screening algorithm of the STEADI program for Thai elderly in the community. In addition, to predictive validity of each component aforementioned, we also explored possible combinations of the components to maximize screening efficiency.

## Methods

### Participants

This research was approved by the Ethics Committee on Human Research, Faculty of Medicine, Chulalongkorn University (IRB No. 532/61). The researcher collected data from the sample group between October 2018 and December 2019. A 1-year prospective cohort study was conducted in Muang District of Nakhon Ratchasima Province, Thailand. To be eligible, the participants must meet all the following criteria: (1) be 65 years old or older; (2) be able to communicate in Thai language; (3) not blind nor deaf; (4) be functionally independent (scored 4 or greater, assessed by the Barthel Activities of Daily Living or ADL) [22] to warrant completion of the screening procedure; and (5) has no cognitive impairment (scored over 14 in those who did not attend school, or scored over 17 in those who graduated grade 7 and lower, or scored over 22 in those who graduated grade 8 or higher education; assessed by Mini Mental State Examination Thai version or MMSE-Thai 2002) [23]. Sample size was estimated based on the following formula [24]: *n*_control_ = (*Z*^2^_α/2_*P*(1-P))/*d*^2^ and *n*_total_ = *n*_control_/(1-prevalence), where *n*_control_ = number of non-fallers, *n*_total_ = number of total subjects, *P* = expected sensitivity (0.76 for TUG), [10] *d* = Allowable error (0.1), *Z*_α/2_ = standard values for type I error at α level of 0.05 ( 1.96), and prevalence = prevalence of fall among Thai elderlies (0.17) [25]. Taking into account the possible drop-out rate of 10%, the required sample size was 462 participants.

**Fig. 1:**
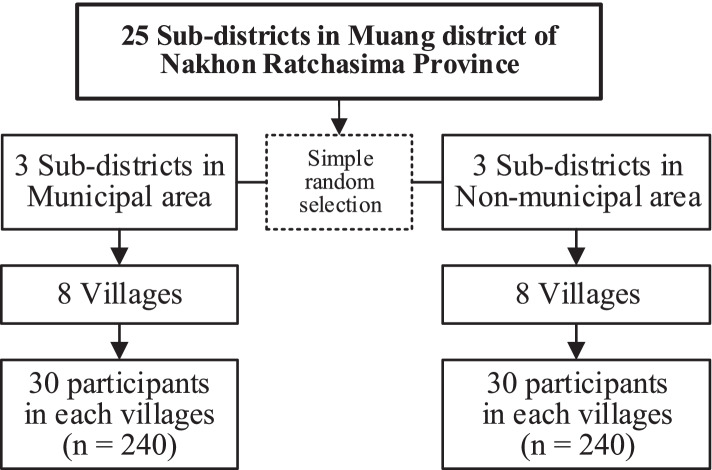
Flow chart of participant selection

To possibly cover the entire range of the target population, multi-stage sampling was utilized in participant selection (Fig. 1). First, six sub-districts were randomly selected (three form urban or municipal areas and the other three from rural or non-municipal areas). Second, eight communities (for urban areas) or villages (for rural areas) were randomly selected for each previously selected sub-district. Third, thirty eligible participants were randomly selected by age-group- and gender-stratification (6 males and 6 females in the 65–69 age group; 3 males and 3 females each in 70–74, 75–79, and 80+ years age groups) and recruited with written informed consent for each community or village, resulting in a total of 480 participants.

### Fall risk screening

Fall risk screening tools/tests used in this study included interview questionnaire (Thai version of Stay Independent Brochure or Thai-SIB and the clinician’s 3 key questions) and physical fitness tests (TUG, 30-S Chair Stand, and The 4-Stage Balance test as recommended by the CDC’s STEADI). The screening questionnaire and the clinician’s 3 key questions were used in the first step, followed by physical fitness tests in the second step.

#### Fall risk assessment questionnaire

The fall risk assessment questionnaire, Thai-SIB, was developed based on the original version of the US CDC’s STEADI program. Standardized procedure including forward-backward translation and cultural adaption was utilized in this questionnaire development (Additional file 1) [26]. Its psychometric properties have been previously assessed [27]. Scoring relied on the number of “yes” answer to each question item, with a total score of 12. Participants are considered to be at-risk of fall based on the following criteria: answer “yes” to 4 or more out of 12 questions, otherwise not at-risk. The clinician’s 3 key questions were also developed by standardized procedure based on the original questions of the STEADI program (fell in the past year?, feel unsteady when standing or walking?, and worries about walking?), with additional detail probing questions for those with previous fall during the past 1 year (number and severity of fall). Participants with a “yes” answer to any of the 3 key questions were considered at-risk of fall, and those with all “no” answeres were considered not at-risk.

#### Physical fitness tests

Three physical fitness tests (TUG, 30-S Chair Stand, and the 4-Stage Balance test) were used in this study based on the CDC STEADI-Algorithm [5].

The TUG is designed to test mobility skills, balance, and fall risk in older persons. The time taken to complete the test is the TUG performance measure, with a longer completion time indicating poorer functional mobility and higher fall risk [28, 29]. We followed the Thai Ministry of Public Health (MOPH) criteria with those taking 10 s or more as being at-risk of fall and not at-risk for those who took less than 10 s [21].

The 30-S Chair Stand assesses lower extremity strength and endurance. The test uses a chair with a straight back without arm rests, and a seat height of 17 inches (43.2 cm). The number of stands less than 5 times is considered at-risk of falling where more than 5 times was considered not at-risk [30].

The 4-Stage Balance test is an assessment of static balance in four different and increasingly challenging positions: (1) feet together, (2) instep of foot advanced to toe of other foot, (3) foot in front of other foot (tandem), and (4) and single-leg stance. Without being able to stand or lasting less than 10 s, all 4 types are considered to be at-risk of falls, standing for 10 s or more is considered not at-risk [31].

#### Data collection

All baseline data collection was conducted at the 6 local sub-district health-promoting hospitals (HPHs) within the study area. The circumstances for assessment, such as floor conditions and chairs, were standardized to minimize the effects from possible confounding variables [29]. A total of 40 research assistants (including 6 registered nurses, 2 physical therapists, and 32 village health volunteers (VHVs) under the jurisdictions of 6 participating sub-district Health Promoting Hospitals or HPHs) with any bachelor degree (or nurse/public health diploma) were recruited and provided with a 3-h training about the study overview and detailed data collection procedure, prior to data collection. Participants were asked to rest fully for 1–2 days, abstain from alcohol for at least 24 h, and visit the nearby HPH on the appointment date in regular clothes and footwear. The data collection date began by the principal investigator (SL) and the registered nurse in the relevant HPH interviewed each participant to (a) collect his/her information about personal demographics (age, gender, educational attainment), health history (underlying diseases such as osteoarthritis, Parkinson disease, stroke, type 2 diabetes), and health related behaviors (cigarette smoking and alcohol consumption), and use of walking aids or assistive device; (b) asked 3 key questions about fall history during the past 1 year; and (c) conduct fall risk screening basing on the Thai-SIB. Physical examination for weight, height, corresponding body mass index (BMI), waist circumference, resting blood pressure, and physical fitness tests were then conducted by two physical therapists. Three physical fitness tests were ordered randomly and conducted.

#### Proposed screening algorithms

**Fig. 2:**
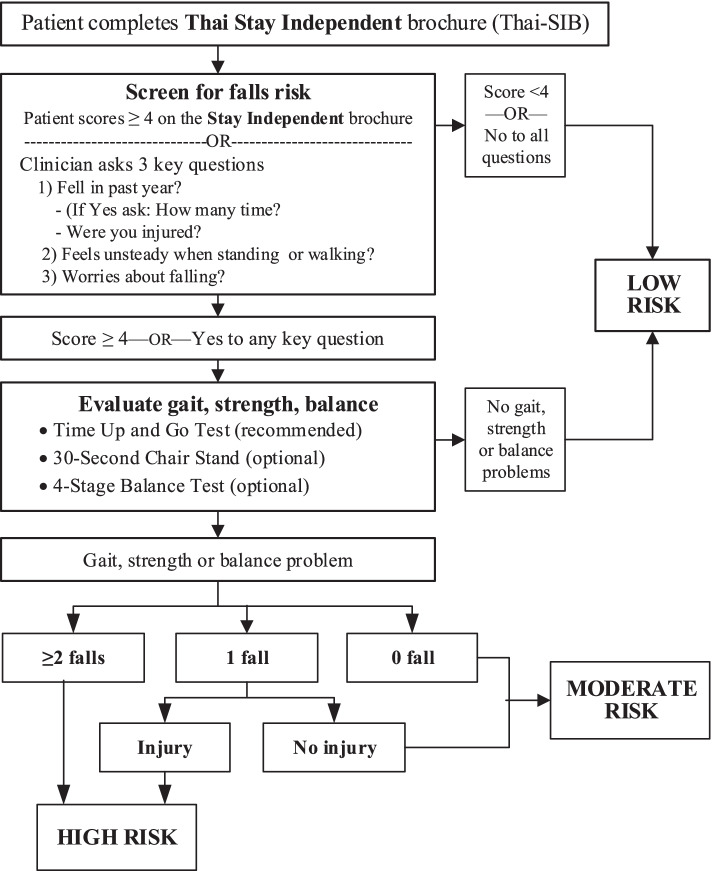
Flow chart of the study. Adapted STEADI-algorithm for determining fall risk level. STEADI, Stopping Elderly Accidents, Deaths, and Injuries

The proposed fall risk screening algorithms followed those used in the US CDC’s STEADI program [5] and was simulated into two-steps (Fig. 2). The screening results from these two steps were utilized in categorizing participants into: **Low risk group**—those who were “not at-risk” from the Step 1, or were “at-risk” from the Step 1 but were “not at-risk” from Step 2; **moderate risk group—**are those who were “at-risk” from both the Steps 1 and 2, together with no history of falling at all in the past 1 year or have had only one fall but no injury; **high-risk group—**are those who were “at-risk” from both the Steps 1 and 2, together with a history of falling with injury or had fallen from 2 or more times during the past one year (Fig. 2 and Additional file 2: Fig. S1).

### Outcome measures

A fall was evaluated according to the definition of the World Health Organization [1] as “an event that results in a person coming to rest inadvertently on the ground or floor or other lower level”. The 1 calendar-year follow-up period of participants started from the day following their baseline data collection date. The first fall event was the primary outcome of interest, predicting the risk of fall. Any later fall events were also counted and treated as repeated outcomes. A self-report form was developed as a structured checklist to collect personal and fall-related information, e.g., date and time of event, location, and details of consequent injuries. Forms were provided and instructed to be completed by the participants or caregivers. Designated VHVs may fill in the form on behave of the participant if needed. VHVs reported all participant’s fall events to the principal investigator monthly. Subsequently, the investigator home visit team investigated the fall events and provided appropriate interventions to prevent future incidents.

### Covariates

In addition to personal demographics, health history, health-related behaviors and home fall safety variables were also considered as potential confounding factors and were assessed by using Thai Home Falls and Accidentals Screening Tool or Thai Home-FAST [32] (this assessment tool was also developed based on standardized procedures including forward-backward translation and cultural adaption). Participants’ home fall hazard assessments were conducted by the principal investigator and two physical therapists one day after finishing their baseline data collection at the HPHs.

#### Data analysis

In describing the participants’ characteristics and baseline fall risk screening results, frequency and percentage were used for categorical data (including gender, age group, marital status, education, underlying disease, smoking, and alcohol consumption), while mean and standard deviation (SD) were used for continuous data with normal distribution (such as body mass index, Thai SIB score, Time Up and Go test, Thai Home-FAST score). Group comparison between fallers and non-fallers was conducted by Fisher’s exact test for categorical data and independent t-test for continuous data with normal distribution.

Screening measures of interest for predictive validity analysis in this study were the 2 individual tests used in Step 1, 3 individual fitness tests used in Step 2, and 6 alternatives of the Step 1 and 2 sequential screening. Cox proportional hazard model was utilized, treating the screening result as the 3-category dummy predictor (low, moderate, and high risk) and first fall occurrence as the binary outcome. Performance of each screening test/alternative was assessed by the AUC, and the corresponding sensitivity and specificity, PPV, and NPV were then estimated. The interpretation of the AUC could be stated as follows: 0.5 = no discrimination, 0.7 to 0.8 = acceptable, 0.8 to 0.9 = excellent, and more than 0.9 = outstanding [33]. Furthermore, discriminative performance of each screening test/alternative were also examined by determining the observed fall probability according to the baseline fall risk level.

Statistical significance level was set at 0.05 for all analyses. STATA Version 15 (StataCorp. 2017. Stata Statistical Software: Release 15. College Station, TX: StataCorp LLC) for Windows was used to perform all data analyses.

## Results

### Fall incidence

During the 12-month follow-up period, 148 out of 480 elderly reported the occurrence of at least one fall incidence, accounting for the cumulative incidence of 30.8 persons (95% CI 26.7, 34.9) per 100 persons per year. The corresponding number of incident falls was 320 during the total follow-up period of 174,354 person-days, resulting in a fall incidence density of 1.84 (95% CI 1.64, 2.05) falls per 1000 person-days. Among those who fell, 47 (31.8%) reported the occurrence of one fall incidence, and 101 (68.2%) recurrent falls. Out of 320 falls, 71 (22.2%) resulted in no injury, 232 (72.5%) mild and moderate injuries, such as contusion, abrasion, knee and leg pain, back pain, and foot injuries, and 17 (5.3%) experienced severe injuries such as hip fracture, arm fracture, leg fracture, and head injuries requiring treatment.

### Baseline characteristic between fallers and non-fallers

The sample comprised of 480 community-dwelling older adults. The mean age was 73.3 ± 6.51 years (range 65–95 years) while 19.2% aged 80 years and older. Almost one third of participants were categorized as fallers (30.8%, 148 out of 480). Two thirds of the fallers were women (66.2%). The mean age of fallers was 74.34 ± 6.36 years (range 65–95 years) while the mean age of non-faller was 72.88 ± 6.54 years (range 65–94 years). Fallers and non-fallers significantly differed according to the composition of gender, marital status, education level, underlying disease including diabetes and dyslipidemia, smoking, and drinking behavior. They did not differ in terms of age and body mass index (Table 1). Their occupations, income, exercise, housing style (one-story non-elevated house, one-story elevated house, or two or more stories house), residential area (rural versus urban), and home fall hazard score were comparable (data not shown). Compared to non-fallers, fallers however had significantly higher baseline fall risk screening score (Thai-SIB 12 items) and lower physical fitness levels as assessed by the Time Up and Go test, and 30-s Chair Stand (Table 1).

**Table 1:** Baseline characteristics of the participants (*n* = 480)

Characteristics	Fallers (*n* = 148)		Non-fallers (*n* = 332)		*P* value
	*n*	(%)	*n*	(%)	
Gender^a^					
Male	50	(33.8)	178	(53.6)	<0.001
Female	98	(66.2)	154	(46.4)	
Age (year)^a^					0.083
65–69	46	(31.1)	132	(39.8)	
70–74	31	(21.0)	79	(23.8)	
75–79	40	(27.0)	60	(18.1)	
≥ 80	31	(21.0)	61	(18.4)	
Mean (SD)	74.34	(6.36)	72.88	(6.54)	
Marital status^a^					0.038
Single	10	(6.8)	26	(7.8)	
Married	80	(54.0)	215	(64.8)	
Widowed, separated	58	(39.2)	91	(27.4)	
Education^a^					0.005
None	15	(10.1)	17	(5.1)	
Primary school	124	(83.8)	267	(80.4)	
Secondary school and above	9	(6.1)	48	(14.5)	
Underlying disease^a^					
Hypertension	94	(63.5)	182	(54.8)	0.089
Diabetes	50	(33.8)	67	(20.2)	0.002
Dyslipidemia	44	(29.7)	70	(21.1)	0.048
Chronic renal failure	7	(4.7)	7	(2.1)	0.142
Smoking^a^					0.016
Never	117	(79.1)	232	(69.9)	
Former	23	(15.5)	54	(16.3)	
Current	8	(5.4)	46	(13.8)	
Alcohol consumption^a^					0.019
Never	110	(74.3)	208	(62.6)	
Former	25	(16.9)	66	(19.9)	
Current	13	(8.8)	58	(17.5)	
Body mass index (kg/m^2^)^b^					0.509
Mean (SD)	23.38	(4.61)	23.09	(4.32)	
Fall risk screening [mean (SD)]^b^					
Thai-SIB 12 items (14 points)	5.93	(3.06)	1.72	(0.95)	<0.001
Physical fitness tests [Mean (SD)]^b^					
Time Up and Go test (min.)	13.43	(5.45)	11.49	(4.25)	<0.001
30-s Chair Stand^a^					0.025
Less than 5 stand in 30 s	13	(8.8)	10	(3.0)	
≥ 5 stand in 30 s	135	(91.2)	322	(97.0)	
The 4-Stage Balance test^a^					0.123
Did not complete all balance stage	7	(4.7)	6	(1.8)	
Complete all balance stage	141	(95.3)	326	(98.2)	
Home fall hazard assessment [Mean (SD)]^b^					
Thai Home-FAST (29 points)	6.99	(4.04)	6.29	(3.66)	0.065

^a^Fisher’s exact test, ^b^independent *t* test

### Predictive validity of the overall screening tools and algorithms

Results about predictive validity of the tools/procedures used in Steps 1 and 2 as well as the 6 sequential fall risk screening algorithms are shown in Table 2 and Additional file 2: Table S1. Between the two screening tools in the first step, the clinician’s 3 key questions had higher ability identify future fallers, as inferred from its higher sensitivity of 93.9% (95% CI 88.8, 97.2) (Table 2). Contrary to this, the Thai-SIB (12 items) had higher specificity, 88.0% (95% CI 84.0, 91.3).

**Table 2:** Predictive validity of the tools/procedures used in the Steps 1 and 2 and 6 sequential fall risk screening algorithms

Screening tools/procedures	AUC	Cutoff	Sen	Spec	PPV	NPV	Duration (min.)
**STEP 1**							
Clinician’s 3 key questions	0.845	1	93.9	75.0	62.6	96.5	< 1
Thai-SIB 12 items	0.828	4	77.7	88.0	74.2	89.8	< 5
**STEP 2**							
TUG	0.584	10	75.0	41.9	36.5	79.0	<1
30-s-Chair Stand	0.526	^a^	8.8	96.4	52.0	70.3	<1
4-Stage balance test	0.515	^b^	4.7	98.2	53.8	69.8	<2
**Sequential screening**							
**Clinician’s 3 key questions followed by**							
TUG	0.774	^c^	71.6	83.1	65.4	86.8	<2
30-s-Chair Stand	0.539	^c^	8.8	99.1	81.3	70.9	<2
4-Stage balance test	0.521	^c^	4.7	99.4	77.8	70.1	<3
**Thai-SIB 12 items followed by**							
TUG	0.767	^c^	62.2	91.3	76.0	84.4	<6
30-s-Chair Stand	0.531	^c^	7.4	98.8	73.3	70.5	<6
4-Stage balance test	0.516	^c^	4.1	99.1	66.7	69.9	<7

*Abbreviations*: *AUC* Area Under the Receiver Operating Characteristic (ROC) curve, *CI* confidence interval, *Sen* sensitivity, *Spec* specificity, *PPV* positive predictive value, *NPV* negative predictive value, *Thai-SIB* Thai Stay independent brochure, *TUG* Time Up and Go test

^a^less than 5 stands in 30 s, ^b^did not complete all balance stage, ^c^a positive test from all tools

In the second step, among the individual physical tests, TUG had the highest ability to identify future fallers, with the sensitivity of 75.0% (95% CI 67.2, 81.7) (Table 2). The remaining two screening procedures including 30-s-chair stand and 4-stage balance test had very low ability to identify future fallers, with the sensitivity of only 8.8% (95% CI 4.8, 14.6) and 4.7% (95% CI 1.9, 9.5), respectively (Table 2). Compared to Step 1, all screening procedures in Step 2 had lower sensitivity.

Validity results of the 6 possible algorithms of the sequential Steps 1 and 2 screenings are shown on the lower portion of Table 2. Compared to the sole screening procedures in Step 1, all of these sequential screening algorithms had lower sensitivity, while their false positivity were slightly improved (lower).

The overall performance of the sequential screening algorithms were examined by dividing the participants into low, moderate, and high fall risk groups and proportional hazard modeling was conducted (Table 3). Result showed that the moderate and high-risk groups had significantly higher hazard ratios than the low-risk group with obvious dose-response patterns for almost all alternatives. These were particularly pronounced for the clinician’s 3 key questions & TUG and the Thai-SIB 12 items & TUG alternatives (Table 3). However, when categorizing risk based on the clinician’s 3 key questions and history of fall in the past one year, or simply basing on the number of positive responses of the clinician’s 3 key questions, results showed that their discriminative ability on future fall probability were even better, both in terms of the relative difference in fall probability and HR (Table 3).

**Table 3:** Relationship between the levels of risk from screening according to risk screening algorithm together with fall history in the past 1 year and chance of falling among elderly

Fall risk screening algorithms	Overall	Faller	Non-faller	Crude HR	95% CI	Adjusted HR^**a**^	95% CI	***P*** value
		***n*** (%)	***n*** (%)					
Clinician’s 3 key questions (basing on the number of positive responses)^b^								
0 point	258	9 (3.5)	249 (96.5)	1.00	Reference	1.00	Reference	
1 point	57	13 (22.8)	44 (77.2)	7.29	3.12, 17.06	6.92	2.92, 16.40	<0.001
≥2 points	165	126 (76.4)	39 (23.6)	40.19	20.36, 79.31	40.35	20.28, 80.29	<0.001
Clinician’s 3 key questions follow by history about the number and severity of previous fall								
Low risk	258	9 (3.5)	249 (96.5)	1.00	Reference	1.00	Reference	
Moderate risk	131	61 (46.6)	70 (53.4)	17.71	8.79, 35.68	18.32	9.01, 37.23	<0.001
High risk	91	78 (85.7)	13 (14.3)	52.48	26.18, 105.18	51.41	25.29, 104.50	<0.001
Clinician’s 3 key questions & TUG								
Low risk	318	42 (13.2)	276 (86.8)	1.00	Reference	1.00	Reference	
Moderate risk	91	46 (50.6)	45 (49.4)	4.72	3.10, 7.18	4.75	3.08, 7.32	<0.001
High risk	71	60 (84.5)	11 (15.5)	11.82	7.92, 17.65	10.43	6.85, 15.90	<0.001
Clinician’s 3 key questions & 30-s-Chair Stand								
Low risk	464	135 (29.1)	329 (70.9)	1.00	Reference	1.00	Reference	
Moderate risk	10	7 (70.0)	3 (30.0)	2.93	1.37, 6.27	3.02	1.36, 6.70	0.006
High risk	6	6 (100.0)	0	7.97	3.47, 18.30	4.49	1.86, 10.83	<0.001
Clinician’s 3 key questions & 4-Stage balance test								
Low risk	471	141 (29.9)	330 (70.1)	1.00	Reference	1.00	Reference	
Moderate risk	6	4 (66.7)	2 (33.3)	2.40	0.89, 6.49	2.36	0.79, 7.08	0.124
High risk	3	3 (100.0)	0	5.68	1.80, 17.94	3.12	0.93, 10.46	0.066
Thai-SIB 12 items & TUG								
Low risk	359	56 (15.6)	303 (84.4)	1.00	Reference	1.00	Reference	
Moderate risk	69	40 (58.0)	29 (42.0)	5.12	3.41, 7.70	4.80	3.16, 7.29	<0.001
High risk	52	52 (100.0)	0	16.03	10.76, 23.87	14.23	9.25, 21.88	<0.001
Thai-SIB 12 items & 30-s-Chair Stand								
Low risk	465	137 (29.5)	328 (70.5)	1.00	Reference	1.00	Reference	
Moderate risk	9	5 (55.6)	4 (44.4)	2.20	0.90, 5.36	1.99	0.80, 4.95	0.137
High risk	6	6 (100.0)	0	7.83	3.41, 18.00	4.49	1.86, 10.83	0.001
Thai-SIB 12 items & 4-Stage balance test								
Low risk	471	142 (30.2)	329 (69.8)	1.00	Reference	1.00	Reference	
Moderate risk	6	3 (50.0)	3 (50.0)	1.79	0.57, 5.61	1.37	0.42, 4.45	0.598
High risk	3	3 (100.0)	0	5.63	1.78, 17.81	3.13	0.93, 10.51	0.065

^a^Adjusted for gender, marital status, education level, diabetes, hyperlipidemia, smoking, alcohol consumption, and home hazard

^b^The clinician’s 3 key questions ask if the elderly ever fell in the past year (yes=2 points); if the elderly feels unsteady when standing or walking (yes=1 point), and if the elderly worries about walking (yes=1 point). The maximum score is 4

### Predictive validity of fall risk categorization

Performance of each step of the sequential screening and assessment was further examined in detail by determining its ability in predicting or identifying future fall risk (for person and event) (Table 4 and Additional file 2: Table S2). Results showed that those who were “not at risk” in Step 1 had a much lower future fall probability than those who were “at risk” irrespective of the assessment result from Step 2. For the Step 1 screening by the clinician’s 3 key questions, the average cumulative fall incidence in the former group ranged between 0 and 3.61 persons per 100 persons per year, while those for the latter group were 55.00 to 81.25 persons per 100 persons per year (Table 4). Proportions of multiple falls were also significantly higher among the “at-risk” (43.69%) than the “not at-risk (1.55%) groups. Within-group comparison between those with versus without gait, strength, or balance problems from the Step 2 assessment did not show any significant difference in the future fall probabilities. These patterns of group differences were also observed when Step 1 was screened by the Thai-SIB (12 items) (Additional file 2: Table S2). Since the numbers of those who were “at-risk” based on the 30-s-Chair Stand and 4-Stage balance test were too small, the following investigation then focused mainly on TUG test results.

**Table 4:** One-year fall incidences among study participants, stratified by Step 1 (the clinician’s 3 key questions) and Step 2 screening results

Risk category	Fall incidence					Number of falls per person						
						0		1		2+		***P*** value†
	***n***	# Fall	IR	(95%CI)	***P*** value†	#	(%)	#	(%)	#	(%)	
**“Not at-risk” from Step 1 screening (*n*= 258)**												
**Timed-Up-and-Go**					1.000							0.670
Not at-risk	116	4	3.45	(0.95, 8.59)		112	(96.55)	3	(2.59)	1	(0.86)	
At-risk	142	5	3.52	(1.15, 8.03)		137	(96.48)	2	(1.41)	3	(2.11)	
**30-s-Chair Stand**					1.000							1.000
Not at-risk	249	9	3.61	(1.67, 6.75)		240	(96.39)	5	(2.01)	4	(1.61)	
At-risk	9	0	0.00	(0.0, 33.63)		9	(100)	0	(0)	0	(0)	
**4-Stage balance test**					1.000							1.000
Not at-risk	254	9	3.54	(1.63, 6.62)		245	(96.46)	5	(1.97)	4	(1.57)	
At-risk	4	0	0.00	(0.0, 60.24)		4	(100.0)	0	(0)	0	(0)	
**Overall**	**258**	**9**	**3.49**	**(1.61, 6.52)**		**249**	**(96.51)**	**5**	**(1.94)**	**4**	**(1.55)**	
**“At-risk” from Step 1 screening (*n*= 222)**												
**Timed-Up-and-Go**					0.163							0.338
Not at-risk	60	33	55.00	(41.61, 67.88)		27	(45.00)	9	(15.00)	24	(40.00)	
At-risk	162	106	65.43	(57.57, 72.72)		56	(34.57)	33	(20.37)	73	(45.06)	
**30-s-Chair Stand**					0.178							0.146
Not at-risk	206	126	61.17	(54.14, 67.86)		80	(38.83)	40	(19.42)	86	(41.75)	
At-risk	16	13	81.25	(54.35, 95.95)		3	(18.75)	2	(12.50)	11	(68.75)	
**4-Stage balance test**					0.489							0.495
Not at-risk	213	132	61.97	(55.09, 68.52)		81	(38.03)	41	(19.25)	91	(42.72)	
At-risk	9	7	77.78	(39.99, 97.19)		2	(22.22)	1	(11.11)	6	(66.67)	
**Overall**	**222**	**139**	**62.61**	**(55.89, 69.00)**	**<0.001**‡	**83**	**(37.39)**	**42**	**(18.92)**	**97**	**(43.69)**	**<0.001‡**

*Abbreviations*: *CI* confidence interval, *IR* incidence rate (number of persons who had fallen per 100 persons per year), *n* number of participants, # number of fall persons or events

^†^Fisher’s Exact test, ^‡^compared between the “Not at-risk” and “At-risk” groups

We further examined the performance of risk categorization basing the number and severity of fall(s) in the previous year among those who were “at risk” from Step 1 screening by the clinician’s 3 key questions (Table 5). Results showed that, compared to those without fall history in the previous year, those who had fallen at least once in the previous year had significantly higher future fall frequency, in terms of both cumulative incidence and the frequency of fall per person; while those who had fallen twice or more in the previous year had significantly higher frequency of fall per person than those with one fall in the previous year. However, among those with one fall in the previous year, future fall frequency did not significantly differ between those with versus without injury, neither in terms of cumulative incidence nor fall frequency per person. These trends were also observed when analyzing among those who were “at-risk” from Step 1 screening by the Thai-SIB 12 items, although less obvious (Additional file 2: Table S3).

**Table 5:** One-year fall incidences (persons per 100 persons per year) according to the number and severity of previous fall among those who were “at risk” from Step 1 screening by the clinician’s 3 key questions, stratified by the Timed-Up-and-Go test result in Step 2 assessment

Risk category	Future fall incidence											
	Cumulative incidence					Number of falls per person						
	n	# Fall	IR	(95%CI)	*P* value†	0		1		2+		*P* value†
						#	(%)	#	(%)	#	(%)	
**Overall**												
**Previous fall history**					<0.001							<0.001
0 fall	114	47	41.23	(32.09, 50.83)		67	(58.77)	11	(9.65)	36	(31.58)	
1 fall, no injury	17	14	82.35	(56.57, 96.20)	a	3	(17.65)	10	(58.82)	4	(23.53)	a
1 fall, injury	53	42	79.25	(65.89, 89.16)	a	11	(20.75)	21	(39.62)	21	(39.62)	a
≥2 falls	38	36	94.74	(82.25, 99.36)	a	2	(5.26)	0	(0)	36	(94.74)	a,b,c
**Total**	**222**	**139**	**62.61**	**(55.89, 69.00)**		**83**	(**37.39**)	**42**	(**18.92**	**97**	(**43.69**)	
**“NOT AT-RISK” from Step 2 assessment**												
**Previous fall history**					<0.001							<0.001
0 fall	33	9	27.27	(13.30, 45.52)		24	(72.73)	1	(3.03)	8	(24.24)	
1 fall, no injury	7	6	85.71	(42.13, 99.64)	a	1	(14.29)	3	(42.86)	3	(42.86)	a
1 fall, injury	12	10	83.33	(51.59, 97.91)	a	2	(16.67)	5	(41.67)	5	(41.67)	a
≥2 falls	8	8	100.0	(63.06, 100.0)	a	0	(0)	0	(0)	8	(100.0)	a,b,c
**Total**	**60**	**33**	**55.00**	**(41.61, 67.88)**		**27**	(**45.00**)	**9**	(**15.00**)	**24**	(**40.00**)	
**“AT-RISK” from Step 2 assessment**												
**Previous fall history**					<0.001							<0.001
0 fall	81	38	46.91	(35.73, 58.33)		43	(53.09)	10	(12.35)	28	(34.57)	
1 fall, no injury	10	8	80.00	44.39, 97.48)		2	(20.00)	7	(70.00)	1	(10.00)	a
1 fall, injury	41	32	78.05	(62.39, 89.44)	a	9	(21.95)	16	(39.02)	16	(39.02)	a
≥2 falls	30	28	93.33	(77.93, 99.18)	a	2	(6.67)	0	(0)	28	(93.33)	a,b,c
**Total**	**162**	**106**	**65.43**	**(57.57, 72.72)**	**0.376‡**	**56**	**(34.57)**	**33**	**(20.37**	**73**	**(45.06)**	**0.338‡**

*Abbreviations*: *CI* confidence interval, *IR* incidence rate (number of persons who had fallen per 100 persons per year), *n* number of participants, # number of fall persons or events

^a^differ from the “0 fall” category with *p*<.05; ^b^differ from the “1 fall, no injury” category with *p*<.05; ^c^differ from the “1 fall, injury” category with *p*<.05

^†^Fisher’s Exact test; ^‡^Compared between the “Not at-risk” and “At-risk” groups

## Discussion

This study showed that, in general, the fall risk sequential screening algorithms proposed by the US CDC in the STEADI program were well applicable in the Thai context. The results largely conformed with the official STEADI screening/assessment guideline, particularly about the suggested choices of screening/assessment tools/procedures used in Step 1 and 2 screening and the overall validity of the algorithms in predicting future fall risk. However, there were two discrepancies between our study result and the STEADI guideline concerning risk categorization after Steps 1 and 2 screening/assessment. Whether these discrepancies were reflective of fact or chance findings requires further investigation.

First conformity: choice of tool used in Step 1 screening. Our results demonstrated that the set of clinician’s 3 key questions is powerful and sufficient to identify future fallers who would benefit from fall preventive interventions. Its sensitivity is better than the Thai-SIB (12 items), which may be due to the higher cut-off of the latter tool. Its better sensitivity than the physical fitness tests (used in Step 2 screening) might relate to its more comprehensive consideration of broader intrinsic fall risk factors. These results also align with prior studies by Lusardi et al. [12], Hesel et al. [34], and Nithman and Vincenzo [20]. When adverse risks of conducting TUG were predicted, either the clinician’s 3 key questions or Thai-SIB (12 items) may be used instead. Due to the high likelihood of serious health, social, and economic consequences of fall in older adults, high sensitivity of the clinician’s 3 key questions is therefore of clinical significance. Its brevity is also practical for utilization in primary care or busy clinical practice.

Second conformity: choice of physical fitness used in Step 2 screening. Our reported markedly high sensitivity of TUG compared to the 30-s-Chair Stand and 4-Stage balance test were also in agreement with the STEADI’s guideline in recommending the TUG as the first choice of physical fitness test, while the other two tests were optional. This was also supported by Lusardi et al.’s report of high post-test probability of the TUG over the Five Times Sit-to-Stand Test (which is comparable to 30-s-Chair Stand) and single-limb stance eyes open, which is a part of the 4-stage balance test in predicting fall risk [12]. However, this was contrary to Nithman and Vincenzo who reported slightly higher sensitivities of 30-s-Chair Stand and 4-stage balance test compared to TUG [20].

Third conformity: the overall validity of the algorithms in predicting future fall risk. Our reported high predictive validity of the sequential screening (composing the clinician’s 3 key questions or SIB in Step 1 screening and TUG in Step 2 assessment) with pronounced dose-response relationship between baseline fall risk level and future fall probability was also consistent with previous reports [18–20]. However, our reported AUCs (0.774 and 0.767), sensitivities (71.6 and 62.2%), and specificities (83.1 and 91.3%) for these two algorithms were higher than those reported previously.

Concerning the two discrepancies, the first one was about the categorization of risk based on Step 1 screening and Step 2 physical fitness results. According to STEADI’s guideline, those who test positive from Step 1 can be categorized into low or moderate risk depending on the physical fitness test result in Step 2, that is, those without evidence of gait, strength, or balance problems will be categorized as “low risk” and otherwise as “moderate risk.” Our findings (Table 4) however showed that compared to those who were negative from Step 1 screening, the probability of future fall was significantly increased for those who were positive irrespective of the test result from the Step 2 assessment. In contrary, probabilities of future fall according to the physical fitness test results did not significantly differ when considering them in the same category of Step 1 screening results. This finding therefore suggested that those who were positive from Step 1 screening should be categorized at the least as “moderate of high risk,” as proposed by Lohman et al. in their investigation about predictive validity and adaptability of the STEADI algorithm to survey data of five annual rounds (2011–2015) of the National Health and Aging Trends Study (NHATS) [19].

The second discrepancy was about the risk categorization based on the number and severity of previous falls. According to STEADI’s guideline, among the individuals who tested positive from Step 1 screening and had evidence of gait, strength, or balance problems in Step 2 assessment, those with no previous fall or had one non-injurious previous fall during the last year are categorized as “moderate risk,” while those with one injurious fall or two or more previous falls during the last year are categorized as “high risk.” In this study, we found that regardless of physical fitness test results, the probability of future fall among those with one previous fall differed significantly from those without previous fall, while these probabilities did not significantly differ for those with one non-injurious versus one injurious fall (Table 5). In addition, the probability of future falls of those with two or more previous fall differed significantly from those with one previous fall.

These two discrepant findings suggested deploying the clinician’s 3 key questions, together with details of the previous fall(s). Risk category may also be reclassified into “low risk” for those who answer “no” to any key question or having SIB score < 4 (with our reported probability or average 1-year incidence of future fall of 3.5%). For those who answer “yes” to any key question or having the SIB score of ≥4, they can be classified as “moderate risk” if no history of fall in the last year (with reported average 1-year incidence of fall of 25–50%). “High risk” classification can be made if individuals have history of one fall during the last year (with average reported 1-year incidence of fall of 70–80%) and “very high risk” classification if having two or more falls during the last year (with average reported 1-year incidence of fall of +90%).

Our study was however conducted only in one geographical location and the sample size was rather limited. In addition, these findings may be culturally specific since older adults in Thailand usually live with family caretakers [6]. They therefore tend to limit their movement and rely on the help of caretakers whenever their physical fitness levels are reduced, resulting in lower than expected probability of future fall risk among those with gait, strength, or balance problems in the Step 2 assessment. These issues therefore need further investigation to acquire firmer evidence prior to inputting them for the consideration in the fall risk assessment guideline adaptation.

This is not to say that physical fitness tests are useless and have no role in the fall risk screening. They can still be utilized as parts of a multifactorial assessment to identify the root cause(s) of the individual’s fall risk or in detecting older individuals who require intervention to mitigate their gait, balance, or strength problems to promote better mobility and consequently improving quality of life.

### Limitations of the study

This study was among the first to investigate the applicability of the US CDC’s STEADI screening algorithms outside the USA. Its prospective cohort design with monthly outcome tracking (falls) fostered valid causal inference. Its community-based nature also supported generalizability of the study findings. During our data collection process, the elderly who were too frail to complete the questionnaire and/or the 3 physical fitness tests were excluded from the study, thus our findings may not be generalized to all the elderly population, i.e., not for frail sub-group. The ceiling effect of the 30-s Chair Stand and 4-stage balance tests may also have occurred due to sample selection bias of the fit elderly; adding these tests to the screening algorithms could potentially decrease the sensitivity and specificity. During follows-up, fall preventive advice provided to those who had fallen might have modified the baseline fall risk for such individuals and introduced biased results to later fall events. Further studies are needed before firm generalizability of the study findings to other populations can be made.

## Conclusions

Our study showed that sequential fall risk screening algorithms of the US CDC’s STEADI program was applicable to the Thai context. Results however suggested that screening algorithms that rely solely on the clinician’s 3 key questions or SIB questionnaire and information about the number and severity of fall in the last year had sufficiently high predictive validity in detecting older adults with high future fall risk. Modification of baseline fall risk categorization may be needed as follows: “low risk” for those who answer “no” to any key question or having SIB score < 4; “moderate risk” for those who answered “yes” to any key question or having the SIB score of ≥4 and no history of fall in the last year; “high risk” if having history of one fall during the last year; and “very high risk” if having two or more falls during the last year. Further studies in other populations with sufficient large sample size are needed before the validity of these findings can be confirmed.

### Policy recommendations and practice implications

1. Screening algorithm that relies solely on the clinician’s 3 key questions or SIB questionnaire and information about the number and severity of fall in the last year can be validly used in detecting older adults with high future fall risk in Thailand.
2. Some modification in the fall-risk categorization is needed: those with one injurious fall should be reclassified into the same category with those with one non-injurious fall during the last year as “high-risk,” while those with two or more falls during the last year should be separately reclassified into the additional category as “very high-risk.”
3. Time-Up-and-Go physical fitness test should be utilized as parts of a multifactorial assessment to identify the root cause(s) of the individual’s fall risk, rather than as tools in the Step 2 assessment.

## Supplementary Information

**Additional file 1.** Concise information about Thai version of Stay Independent Brochure (Thai-SIB).

**Additional file 2: Figure S1.** Two-steps fall risk categorization (Thai SIB=Thai version of the Stay Independent brochure). **Table S1.** Predictive validity of the tools/procedures used in the Steps 1 and 2 and 6 sequential fall risk screening algorithms. **Table S2.** One-year fall incidence s among study participants, stratified by the Step 1 (the Thai-SIB 12 items) and Step 2 screening results. **Table S3.** One-year fall incidences (persons per 100 person per year) according to the number and severity of previous fall among those who were “at risk” from Step 1 screening by the Thai-SIB 12 items, stratified by the Timed-Up-and-Go test result in Step 2 assessment.

## Data Availability

Additional data are available from the corresponding author upon request.

## Abbreviations

AUC: Area Under the Receiver Operating Characteristic (ROC) curve
CDC: Centers for Disease Control and Prevention
CI: Confidence interval
HPH: Health Promoting Hospital
HR: Hazard ratio
IR: Incidence rate
NPV: Negative predictive value
PPV: Positive predictive value
SD: Standard deviation
Sen: Sensitivity
Spec: Specificity
STEADI: Stopping Elderly Accidents, Deaths & Injuries
Thai Home-FAST: Thai Home Falls and Accidentals Screening Tools
Thai-SIB: Thai Stay Independent Brochure
TUG: Timed Up and Go test
VHV: Village health volunteers
