# Supplementary material for: Predictive validity of the Stopping Elderly Accidents, Deaths & Injuries (STEADI) program fall risk screening algorithms among community-dwelling Thai elderly
Source: BMC Med. 2022 Mar 14;20:78. doi: 10.1186/s12916-022-02280-w (PMC8919544; doi:10.1186/s12916-022-02280-w)
Supplement: Supplementary file 1 — Additional file 1. Concise information about Thai version of Stay Independent Brochure (Thai-SIB). [file 12916_2022_2280_MOESM1_ESM.docx]

**Additional file 1**

Concise information about Thai version of Stay Independent Brochure (Thai-SIB)

The Stopping Elderly Accidents, Deaths, and Injuries (STEADI) initiative was created by the Injury Center of US Centers for Disease Control and Prevention, as a multifactorial approach to fall prevention. The STEADI initiative aims to reduce fall among older US adults living in community.^[[1]](#footnote-1)^ It offers a coordinated approach to implementing the American and British Geriatrics Societies’ Clinical Practice Guideline for fall prevention. Three core elements of the STEADI include:

(1) Screen patients for fall risk,

(2) Assess modifiable risk factors, and

(3) Intervene to reduce risk by using effective clinical and community strategies.

Stay Independent Brochure (SIB) is a short self-assessment questionnaire, comprised of 12 questions.^[[2]](#footnote-2)^ The SIB is used in “Step (1) Screen patients for fall risk”, in accompanied with the clinician’s 3 key questions which are about the elderly’s fall history in the past year. The SIB’s 12 questions are as follow;

| **Item** | **Question** |
| --- | --- |
| 1 | I have fallen in the past year |
| 2 | I use or have been advised to use a cane or walker to get around safely. |
| 3 | Sometimes I feel unsteady when I am walking. |
| 4 | I steady myself by holding onto furniture when walking at home. |
| 5 | I am worried about falling. |
| 6 | I need to push with my hands to stand up from a chair. |
| 7 | I have some trouble stepping up onto a curb. |
| 8 | I often have to rush to the toilet. |
| 9 | I have lost some feeling in my feet. |
| 10 | I take medicine that sometimes makes me feel light-headed or more tired than usual. |
| 11 | I take medicine to help me sleep or improve my mood. |
| 12 | I often feel sad or depressed. |

Each “Yes” answer counts as one point.The individual who scores 4 points or more is considered at risk for falling and needs further assessment in Step 2.

Thai version of Stay Independent Brochure (Thai SIB) was developed by Loonlawong et al., based on the original US CDC’s SIB and related literature, for utilizing as the first step fall risk screening among older adults living in community in Thailand.^[[3]](#footnote-3)^ Standardized procedure, including forward- backward translation and cultural adaption, was utilized in the development of Thai SIB. There are two versions of Thai SIB: the original (12 question items) and the modified (18 question items). In terms of psychometric properties, both Thai SIB versions had good content validity (IOC: 0.80 to 1.00), and excellent interclass correlation coefficient (ICC) of test-retest and inter-rater reliability for both SIB versions (ICC 0.89-0.95). The coefficient alpha was higher than the recommended value of 0.70 for the total score of both SIB versions, and the convergent validity between the Timed Up and Go (TUG) and Berg Balance Scale (BBS) tests was statistically significant (p<0.001).

1. Johnston YA, Bergen G, Bauer M, Parker EM, Wentworth L, McFadden M, Reome C, Garnett M. Imp8lementation of the Stopping Elderly Accidents, Deaths, and Injuries Initiative in Primary Care: An Outcome Evaluation. Gerontologist. 2019 Nov 16;59(6):1182-1191. doi: 10.1093/geront/gny101. [↑](#footnote-ref-1)
2. Rubenstein LZ, Vivrette R, Harker JO, Stevens JA, Kramer BJ. Validating an evidence-based, self-rated fall risk questionnaire (FRQ) for older adults. J Safety Res. 2011;42(6):493–499. doi:10.1016/j.jsr.2011.08.006 [↑](#footnote-ref-2)
3. Loonlawong S, Limroongreungrat W, Jiamjarasrangsi W. The Stay Independent Brochure as a Screening Evaluation for Fall Risk in an Elderly Thai Population. Clin Interv Aging. 2019 Dec 12;14:2155-2162. doi: 10.2147/CIA.S233414. eCollection 2019. [↑](#footnote-ref-3)
