# Supplementary material for: Predictive validity of the Stopping Elderly Accidents, Deaths & Injuries (STEADI) program fall risk screening algorithms among community-dwelling Thai elderly
Source: BMC Med. 2022 Mar 14;20:78. doi: 10.1186/s12916-022-02280-w (PMC8919544; doi:10.1186/s12916-022-02280-w)
Supplement: Supplementary file 2 — Additional file 2: Figure S1. Two-steps fall risk categorization (Thai SIB=Thai version of the Stay Independent brochure). Table S1. Predictive validity of the tools/procedures used in the Steps 1 and 2 and 6 sequential fall risk screening algorithms. Table S2. One-year fall incidence s among study participants, stratified by the Step 1 (the Thai-SIB 12 items) and Step 2 screening results. Table S3. One-year fall incidences (persons per 100 person per year) according to the number and severity of previous fall among those who were “at risk” from Step 1 screening by the Thai-SIB 12 items, stratified by the Timed-Up-and-Go test result in Step 2 assessment. [file 12916_2022_2280_MOESM2_ESM.docx]

**STEP 1 SCREENING**

**Thai SIB**

Score < 4

Score > 4

**3 key questions**

“No” to all

“Yes” to any question

**STEP 2 ASSESSMENT**

**or**

- **Time Up and Go Test (recommended)**
- **30-Second Chair Stand (optional)**
- **4-Stage Balance Test (optional)**

No

gait,

strength

or

balance problems

Gait,

strength

or

balance

problems

0 fall

1 fall

> 2 fall

No injury

**Number**

**(and severity)**

**of fall in the past year**

injury

**RISK CATEGORIZATION**

**LOW**

**MODERATE**

**HIGH**

**Figure S1** Two-steps fall risk categorization (Thai SIB=Thai version of the Stay Independent brochure)**.**

**Table S1** Predictive validity of the tools/procedures used in the Steps 1 and 2 and 6 sequential fall risk screening algorithms

| Screening tools/ procedures | AUC  (95% CI) | Cutoff | %Sensitivity  (95% CI) | %Specificity  (95% CI) | %PPV  (95% CI) | %NPV  (95% CI) | %FP | %FN | Duration (mean) |
| --- | --- | --- | --- | --- | --- | --- | --- | --- | --- |
| **STEP 1** |  |  |  |  |  |  |  |  |  |
| Clinician’s 3 key questions | 0.845 (0.814, 0.874) | 1 | 93.9 (88.8, 97.2) | 75.0 (70.0, 79.6) | 62.6 (55.9, 69.0) | 96.5 (93.5, 98.4) | 25.0 | 6.1 | < 2 min |
| Thai-SIB 12 items | 0.828 (0.790, 0.866) | 4 | 77.7 (70.1, 84.1) | 88.0 (84.0, 91.3) | 74.2 (66.6, 80.9) | 89.8 (86.0, 92.9) | 12.0 | 22.3 | < 5 min |
| **STEP 2** |  |  |  |  |  |  |  |  |  |
| TUG | 0.584 (0.540, 0.628) | 10 | 75.0 (67.2, 81.7) | 41.9 (36.5, 47.4) | 36.5 (31.1, 42.2) | 79.0 (72.2, 84.7) | 58.1 | 25.0 | < 1 min |
| 30s- Chair Stand | 0.526 (0.501, 0.551) | † | 8.8 (4.8, 14.6) | 96.4 (93.8, 98.1) | 52.0 (31.3, 72.2) | 70.3 (65.9, 74.5) | 3.6 | 91.2 | < 1 min |
| 4-Stage balance test | 0.515 (0.496, 0.533) | ‡ | 4.7 (1.9, 9.5) | 98.2 (96.1, 99.3) | 53.8 (25.1, 80.8) | 69.8 (65.4, 73.9) | 1.8 | 95.3 | < 2 min |
| **SEQUENTIAL SCREENING** |  |  |  |  |  |  |  |  |  |
| **Clinician’s 3 key questions followed by** | |  |  |  |  |  |  |  |  |
| TUG | 0.774 (0.732, 0.815) | ¥ | 71.6 (63.6, 78.7) | 83.1 (78.7, 87.0) | 65.4 (57.6, 72.7) | 86.8 (82.6, 90.3) | 16.9 | 28.4 | < 2 min |
| 30s-Chair Stand | 0.539 (0.516, 0.563) | ¥ | 8.8 (4.8, 14.6) | 99.1 (97.4, 99.8) | 81.3 (54.4, 96.0) | 70.9 (66.5, 75.0) | 0.9 | 91.2 | < 2 min |
| 4-Stage balance test | 0.521 (0.503, 0.538) | ¥ | 4.7 (1.9, 9.5) | 99.4 (97.8, 99.9) | 77.8 (40.0, 97.2) | 70.1 (65.7, 74.2) | 0.6 | 95.3 | < 3 min |
| **Thai-SIB 12 items followed by** | |  |  |  |  |  |  |  |  |
| TUG | 0.767 (0.725, 0.809) | ¥ | 62.2 (53.8, 70.0) | 91.3 (87.7, 94.1) | 76.0 (67.4, 83.3) | 84.4 (80.2, 88.0) | 8.7 | 37.8 | < 6 min |
| 30s-Chair Stand | 0.531 (0.509, 0.553) | ¥ | 7.4 (3.8, 12.9) | 98.8 (96.9, 99.7) | 73.3 (44.9, 92.2) | 70.5 (66.2, 74.6) | 1.2 | 92.6 | < 6 min |
| 4-Stage balance test | 0.516 (0.499, 0.532) | ¥ | 4.1 (1.5, 8.6) | 99.1 (97.4, 99.8) | 66.7 (29.9, 92.5) | 69.9 (65.5, 74.0) | 0.9 | 95.9 | < 7 min |

**Abbreviations:** AUC: Area Under the Receiver Operating Characteristic (ROC) curve; CI: Confidence Interval; PPV: Positive predictive value; NPV: Negative predictive value;

FP: False positive; FN: False negative; Thai-SIB: Thai Stay Independent brochure; TUG: Time Up and Go test; †: Less than 5 stands in 30 seconds;

‡: Did not complete all balance stage; ¥: A positive test from all tools

**Table S2** One-year fall incidence s among study participants, stratified by the Step 1 (the Thai-SIB 12 items) and Step 2

screening results

| **Risk Category** | **Fall Incidence** | | | | | **Number of falls per person** | | | | | | |
| --- | --- | --- | --- | --- | --- | --- | --- | --- | --- | --- | --- | --- |
|  |  |  |  |  |  | **0** | | **1** | | **2+** | | **p-value†** |
|  | **n** | **# Fall** | **IR** | **(95%CI)** | **p-value†** | **#** | **(%)** | **#** | **(%)** | **#** | **(%)** |  |
|  | **“Not at-risk” from Step 1 screening (n=325)** | | | | | | | | | | | |
| **Timed-Up-and-Go** |  |  |  |  | 1.000 |  |  |  |  |  |  | 0.922 |
| Not at-risk | 142 | 14 | 9.86 | (5.50, 15.99) |  | 128 | (90.14) | 7 | (4.93) | 7 | (4.93) |  |
| At-risk | 183 | 19 | 10.38 | (6.37, 15.74) |  | 164 | (89.62) | 11 | (6.01) | 8 | (4.37) |  |
| **30s- Chair Stand** |  |  |  |  | 0.269 |  |  |  |  |  |  | 0.167 |
| Not at-risk | 315 | 31 | 9.84 | (6.79, 13.68) |  | 284 | (90.16) | 16 | (5.08) | 15 | (4.76) |  |
| At-risk | 10 | 2 | 20.0 | (2.52, 55.61) |  | 8 | (80.0) | 2 | (20.0) | 0 | (0) |  |
| **4-Stage balance test** |  |  |  |  | 0.350 |  |  |  |  |  |  | 0.350 |
| Not at-risk | 321 | 32 | 9.97 | (6.92, 13.78) |  | 289 | (90.03) | 17 | (5.30) | 15 | (4.67) |  |
| At-risk | 4 | 1 | 25.0 | (0.63, 80.59) |  | 3 | (75.0) | 1 | (25.0) | 0 | (0) |  |
| **Overall** | **325** | **33** | **10.35** | **(7.09, 13.96)** |  | **292** | **(89.85)** | **18** | **(5.54)** | **15** | **(4.62)** |  |
|  | **“At-risk” from Step 1 screening (n=155)** | | | | | | | | | | | |
| **Timed-Up-and-Go** |  |  |  |  | 0.376 |  |  |  |  |  |  | 0.582 |
| Not at-risk | 34 | 23 | 67.65 | (49.47, 82.61) |  | 11 | (32.35) | 5 | (14.71) | 18 | (52.94) |  |
| At-risk | 121 | 92 | 76.03 | (67.43, 83.32) |  | 29 | (23.97) | 24 | (19.83) | 68 | (56.20) |  |
| **30s- Chair Stand** |  |  |  |  | 0.100 |  |  |  |  |  |  | 0.107 |
| Not at-risk | 140 | 104 | 74.29 | (66.22, 81.29) |  | 36 | (25.71) | 29 | (20.71) | 75 | (53.57) |  |
| At-risk | 15 | 11 | 73.33 | (44.90, 92.21) |  | 4 | (26.67) | 0 | (0) | 11 | (73.33) |  |
| **4-Stage balance test** |  |  |  |  | 0.696 |  |  |  |  |  |  | 0.370 |
| Not at-risk | 146 | 109 | 74.66 | (66.80, 81.49) |  | 37 | (25.34) | 29 | (19.86) | 80 | (54.79) |  |
| At-risk | 9 | 6 | 66.67 | (29.93, 92.51) |  | 3 | (33.33) | 0 | (0) | 6 | (66.67) |  |
| **Overall** | **155** | **115** | **74.19** | **(66.56, 80.88)** | **<0.001‡** | **40** | **(25.81)** | **29** | **(18.71)** | **86** | **(55.48)** | **<0.001‡** |

**Abbreviations:** CI: Confidence interval; IR: Incidence rate (number of persons who had fallen per 100 persons per year); n: number of participants;

#: number of fall persons or events; †: Fisher’s Exact test; ‡: Compared between the “Not at-risk” and “At-risk” groups

**Table S3** One-year fall incidences (persons per 100 person per year) according to the number and severity of previous fall among those who were “at risk” from Step 1 screening by the Thai-SIB 12 items, stratified by the Timed-Up-and-Go test result in Step 2 assessment.

| **Risk Category** | **FUTURE FALL INCIDENCE** | | | | | | | | | | | |
| --- | --- | --- | --- | --- | --- | --- | --- | --- | --- | --- | --- | --- |
|  | **Cumulative Incidence** | | | | | **Number of falls per person** | | | | | | |
|  | **n** | **# Fall** | **IR** | **(95%CI)** | **p-value†** | **0** | | **1** | | **2+** | | **p-value†** |
|  |  |  |  |  |  | **#** | **(%)** | **#** | **(%)** | **#** | **(%)** |  |
| **OVERALL** | | | | | | | | | | | | |
| **Previous fall history** |  |  |  |  | <0.001 |  |  |  |  |  |  | <0.001 |
| 0 fall | 81 | 41 | 50.62 | (39.27, 61.91) |  | 40 | (49.38) | 8 | (9.88) | 33 | (40.74) |  |
| 1 fall, no injury | 10 | 10 | 100.0 | (69.15, 100.0) | a | 0 | (0) | 6 | (60.0) | 4 | (40.0) | a |
| 1 fall, injury | 34 | 34 | 100.0 | (89.72, 100.0) | a | 0 | (0) | 15 | (44.12) | 19 | (55.88) | a |
| >2 falls | 30 | 30 | 100.0 | (88.43, 100.0) | a | 0 | (0) | 0 | (0) | 30 | (100.0) | a, b, c |
| **Total** | **155** | **115** | **74.19** | **(66.56, 80.88)** |  | **40** | **(25.81)** | **29** | (**18.71** | **86** | (**55.48**) |  |
| **“NOT AT-RISK” from Step 2 assessment** | | | | | | | | | | | | |
| **Previous fall history** |  |  |  |  | 0.002 |  |  |  |  |  |  | 0.002 |
| 0 fall | 18 | 7 | (38.89) | (17.30, 64.25) |  | 11 | (61.11) | 1 | (5.56) | 6 | (33.33) |  |
| 1 fall, no injury | 4 | 4 | (100.0) | (39.76, 100.0) |  | 0 | (0) | 1 | (25.0) | 3 | (75.00) |  |
| 1 fall, injury | 7 | 7 | (100.0) | (59.04, 100.0) | a | 0 | (0) | 3 | (42.86) | 4 | (57.14) | a |
| >2 falls | 5 | 5 | (100.0) | (47.82, 100.0) | a | 0 | (0) | 0 | (0) | 5 | (100.0) | a |
| **Total** | **34** | **23** | **(67.65)** | **(49.47, 82.61)** |  | **11** | **(32.35)** | **5** | **(14.71)** | **18** | **(52.94)** |  |
| **“AT-RISK” from Step 2 assessment** | | | | | | | | | | | | |
| **Previous fall history** |  |  |  |  | <0.001 |  |  |  |  |  |  | <0.001 |
| 0 fall | 63 | 34 | (53.97) | (40.94, 66.61) |  | 29 | (46.03) | 7 | (11.11) | 27 | (42.86) |  |
| 1 fall, no injury | 6 | 6 | (100.0) | (54.07, 100.0) | a | 0 | (0) | 5 | (83.33) | 1 | (16.67) | a |
| 1 fall, injury | 27 | 27 | (100.0) | (87.23, 100.0) | a | 0 | (0) | 12 | (44.44) | 15 | (55.56) | a |
| >2 falls | 25 | 25 | (100.0) | (86.28, 100.0) | a | 0 | (0) | 0 | (0) | 25 | (100.0) | a, b, c |
| **Total** | **121** | 92 | **(76.03)** | **(67.43, 83.32)** | **0.491‡** | **29** | **(23.97)** | **24** | (**19.83)** | **68** | (**56.20**) | **0.582‡** |

**Abbreviations:** CI: Confidence interval; IR: Incidence rate (number of persons who had fallen per 100 persons per year); n: number of participants; #: number of fall persons or events; a: differ from the “0 fall” category with p<.05; b: differ from the “1 fall, no injury” category with p<.05; c: differ from the “1 fall, injury” category with p<.05;

†: Fisher’s Exact test; ‡: Compared between the “Not at-risk” and “At-risk” groups.
